# Case Report: Early treatment of anterior crossbite in young patients using clear aligners

**DOI:** 10.3389/fdmed.2026.1761514

**Published:** 2026-04-02

**Authors:** Aldo Giancotti, Paola Mozzicato, Martina Carillo, Franceco Pachì

**Affiliations:** Department of Clinical Sciences and Translational Medicine, University of Rome Tor Vergata, Roma, Italy

**Keywords:** anterior crossbite, clear aligner, digital plan, growing patients, pseudo-Class III

## Abstract

**Aim:**

The aim of this paper is to describe the effects of an alternative treatment for anterior single-tooth crossbite in young patients with dental Class I and/or pseudo-Class III malocclusions. Indeed, anterior crossbite malocclusion of one or more teeth, without posterior crossbite association, is a relatively frequent condition in mixed dentition. This type of malocclusion is usually treated with either fixed or removable appliances. Currently, several clinical studies indicate the use of clear aligners as an effective option. Hence, clear appliances in early treatment could be a valid alternative to traditional removable and fixed orthodontic devices.

**Materials and methods:**

Eighteen growing patients with dental Class I and/or pseudo-Class III malocclusion, all presenting anterior crossbite, including 11 males and 7 females aged 8.3 to 9.9, had been treated with aligners between 2018 and 2021 in order to solve anterior cross bite of upper central incisors. Selected from the aforementioned sample, two pediatric cases - aged 9 - reporting anterior single-tooth crossbite malocclusion are further described in the Clinical Reports section.

**Results:**

In all treated patients, anterior crossbite was resolved within 12 months of treatment using clear aligners only, as exemplified by the two cases illustrated herein. By the end of clear aligner-based treatment, in all the 18 cases positive cephalometric and dental changes were observed, as well as normalized overjet and overbite and preservation of periodontal tissues.

**Conclusion:**

As confirmed by the results achieved, clear aligner treatment can be considered as a valid approach to rapidly correct anterior crossbite in growing patients, as long as a proper diagnostic procedure and optimal patient compliance level are ensured.

## Introduction

Anterior crossbite is defined as a reverse sagittal relationship between maxillary and mandibular incisors. Clinically, there are three types of anterior crossbite: dental, skeletal and functional (1, 2). Anterior dental crossbite is characterized by an incorrect inclination of the incisors. Anterior skeletal crossbite may also comprise a basal bone discrepancy in the sagittal plane and is often associated with true skeletal Class III. Finally, the functional type is considered to be a positional form resulting from mesial displacement of the mandible into the anterior position, and is often also referred to as pseudo or apparent.

Moyers defined the mentioned third type as a positional malocclusion with an acquired neuromuscular reflex, and considered the hypothesis that the positional relationship may occur with an early interference with the mandibular closure muscular reflex (3). Patients with a pseudo-Class III malocclusion mainly present a Class I or mild Class III skeletal relationship, whereas the mandible appears as morphologically normal. Several causal factors (dental, functional, skeletal) have been suggested in pseudo-Class III malocclusion with anterior crossbite, including:

- Ectopic eruption of upper central incisors;
- Premature loss of deciduous molars;
- Anomalies in tongue position;
- Neuromuscular features;
- Trasversal maxillary discrepancy.

Anterior crossbite malocclusion, lacking posterior crossbite association, is a relatively incident condition consequent to eruption anomalies that can be detected both in deciduous and mixed dentition. Several clinicians believe in the advantages of early intervention, and have suggested a number of reasons for early correction of anterior crossbite even during deciduous dentition (1, 4–10).

This is indicated to reduce both the need for further orthodontic treatment and the development of a posterior crossbite induced by occlusal interference and anterior shift (11–15).

Anterior crossbite malocclusions are usually treated with either fixed or removable appliances in order to correct occlusal interferences of one or more teeth (2, 6, 11, 14, 16–23).

Over the past few decades, clear aligners have served as an effective alternative to conventional orthodontic therapy (17, 24–26).

In particular, several studies have reported their effectiveness in treating Class III malocclusions, especially in cases of orthodontic camouflage during adulthood (27–29) and in growing patients (30, 31).

In this regard, clear aligner therapy can be considered as a prioritized option not only for clinical reasons, but also depending on the fact that, nowadays, children value the appliance's aesthetics (32).

In addition, aligners ensure better dental hygiene and greater comfort compared to conventional orthodontic devices, improving patients’ satisfaction (33).

Finally, their biomechanics - based on a digital plan - allows for highly accurate control, especially when it comes to predictable movements, such as crown-tipping ones (34, 35).

The aim of this study is to demonstrate the effectiveness of clear aligners in the treatment of growing patients with pseudo-class III malocclusion, reporting the results collected from a sample of 18 patients diagnosed with anterior crossbite. Furthermore, the article includes a brief clinical report with two randomly selected cases from the sample, both featuring a single-tooth anterior crossbite treated by means of clear aligners.

## Materials and method

The herein study was designed as a retrospective observational study to assess the effectiveness of clear aligners as an alternative treatment for the correction of anterior crossbite. The study was completed in 2025 at a private clinical practice based in Rome, Italy, observing a number of clinical cases over a period of three years.

### Study sample

The cases presenting with anterior crossbite were recruited based on a set of inclusion and exclusion criteria.

Specifically, inclusion criteria were as follows:

- Mixed dentition phase;
- Anterior cross bite associated with a Dental Class I and/or pseudo-Class III malocclusion;
- Good compliance.

The exclusion criteria consisted of:

- Previous orthodontic treatment;
- Lack of compliance;
- Transversal skeletal discrepancies;
- Sagittal skeletal discrepancies.

The final sample included 18 enrolled patients, 11 males and 7 females (average age 9.3). Consent forms were duly submitted by all patients’ parents/caregivers. The observed cases had been treated with clear aligner therapy by means of a digital protocol between 2018 and 2021 in order to solve anterior crossbite of upper central incisors. Patients had been instructed to wear aligners for at least 20–22 h per day, with aligner changes scheduled every 5 days.

### Measurement protocol

Based on our study design, clinical records, including intraoral photographs, digital dental models, and lateral cephalograms, were obtained at baseline (T0) and after 12 months of treatment (T1). Specifically, measurements included the following dental and cephalometric values: overjet, arch perimeter, Maxillary intercanine distance, Mandibular intercanine distance, SNA, SNB, ANB, and U1.NA (Table 1).

**Table 1:** Dental and cephalometric values at T0 and T1.

Values	Mean (SD) T0	Mean (SD) T1	*P* Value*
Overjet	−1.10 mm (0.35)	0.25 mm (0.88)	<.001
Arch perimeter	91.80 mm (6.60)	92.10 mm (6.61)	.250
IC (Mx)	41.73 mm (2.12)	43.30 mm (2.71)	.006
IC (Md)	35.70 mm (2.04)	37.30 mm (2.66)	.031
SNA	82.20° (3.74)	83.10° (3.76)	.114
SNB	78.30° (3.76)	79.15° (3.69)	.276
ANB	3.90° (1.74)	3.95° (2.48)	.589
U1-NA	20.60° (4.88)	23.87°(4.67)	.002

Arch perimeter indicates maxillary arch perimeter; IC (Mx) indicates intercanine distance in the maxilla; IC (Md) indicates intercanine distance in the mandible;SD indicates standard deviation; T0 indicates time prior to treatment; T1 indicates time after 12 months of active treatment.

*Paired *t-*test. Significance level of *P* < .05.

All the dental measurements were calculated exporting STL files of digital models at T0 and T1 and using 3Shape Ortho System 2023.1 software. The overjet increase was measured in millimeters and corresponded to the difference of overjet between T0 and T1 (Table 1).

Evaluation of the maxillary arch perimeter was performed by measuring values on an initial and final digital model, starting from the mesial surface of the permanent first molar, passing around the arch over the contact points of the posterior teeth and incisal edges of the anterior teeth to the mesial surface of the permanent first molar on the opposite side. The increase in arch perimeter was calculated by measuring the difference between the perimeter of the arch at T0 and T1 (Table 1). The arch perimeter corresponded to the sum of the following five segments: the distance from the mesial point of the first molars to the distal point of the canines, the distance from the distal point of the canines to the distal point of the central incisors on both sides, and finally, the distance between the distal points of the right and left central incisors.

The intercanine distance in the maxilla and mandible was calculated by measuring the difference between the intercanine distances at T0 and T1 (Table 1).

The cephalometric angles evaluated were SNA, SNB, and ANB, serving as a means to assess the position of the maxilla and mandible relative to the cranial base, as well as the position of the maxilla and mandible to one another. The upper incisor inclination (U1-NA) was also evaluated compared to the NA line (Nasion-A point). The change in cephalometric angles was determined by the difference in the values between T0 and T1, measured by using the Delta-Dent software, Outside Format (Milan, Italy) (Table 1).

### Statistical analysis

A statistical analysis was conducted using the Statistical Package for the Social Sciences version 17.0 of the Shapiro–Wilk test, demonstrating that the data was normally distributed. Therefore, parametric tests were carried out. The data analysis included descriptive tests (Chi-square and Student's t-test) to characterize the sample. Paired t-tests were used to evaluate the effects (changes occurring during treatment, T1−T0) of the treatment protocol for correcting anterior crossbite. A Student's t-test was used to compare the changes occurring during treatment (T1−T0). The level of significance was set at *P* < 0.05. Values resulted as being statistically significant.

Herein, the treatment of two randomly selected cases, aged 9, is further described in the case report section. Both patients reported pseudo-Class III malocclusion and anterior crossbite of an upper central incisor. They were overseen with the objective of solving their clinical condition. Our diagnosis was based on clinical examination, digital models, panoramic and lateral x-ray records.

The functional mandibular shift caused by anterior crossbite was considered in order to diagnose the actual mandibular position and to establish the proper condyle positioning into a centric relationship. In order to solve this type of malocclusion, three possible orthodontic approaches were taken into account:

- fixed appliance treatment;
- conventional removable appliance treatment;
- clear aligner treatment.

The decision-making process led to the use of clear aligners, driven by three main reasons:

1. Having the possibility of using a dedicated software (ClinCheck Pro® register) that would allow for visualization of the resolution, possibility of sharing insights with parents/caregivers, and raising awareness;
2. Allowing for optimal resolution of the anterior crossbite and reciprocal correction of upper and lower incisor position;
3. Benefiting from a potentially more rapid resolution thanks to the presence of a pair of aligners that would reduce occlusal interference.

## Results

In all treated cases, as exemplified by the case reports illustrated hereinafter, treatment objectives were achieved in a limited amount of time, leading to full satisfaction of both patients and caregivers. The overall active treatment duration did not exceed 12 months, raising no relevant concerns nor disruptions. Moreover, proper hygiene was maintained, also allowing for gingival recession resolution in three of the eighteen cases. Regarding compliance, all patients were highly compliant by wearing and cleaning aligners according to medical prescription. In all cases, anterior crossbite was resolved, and posterior intercuspation was properly maintained.

The average age of the treated sample was 9.07 years (±0.79). Cumulative characteristics of enrolled patients are shown in Table 1. By comparing initial and final records (T0−T1), the positive effects of treatment protocols for anterior crossbite correction can be observed. Indeed, the study group showed a significant increase in overjet (*P* < .001), maxillary intercanine distance (*P* = .006), mandibular intercanine distance (*P* = .031), and upper incisor inclination (*P* = .002).

### Clinical reports

The following clinical reports aim at showing the treatment procedures and outcomes in two randomly selected patients among the observed sample.

### Case report 1

**Figure 1:**
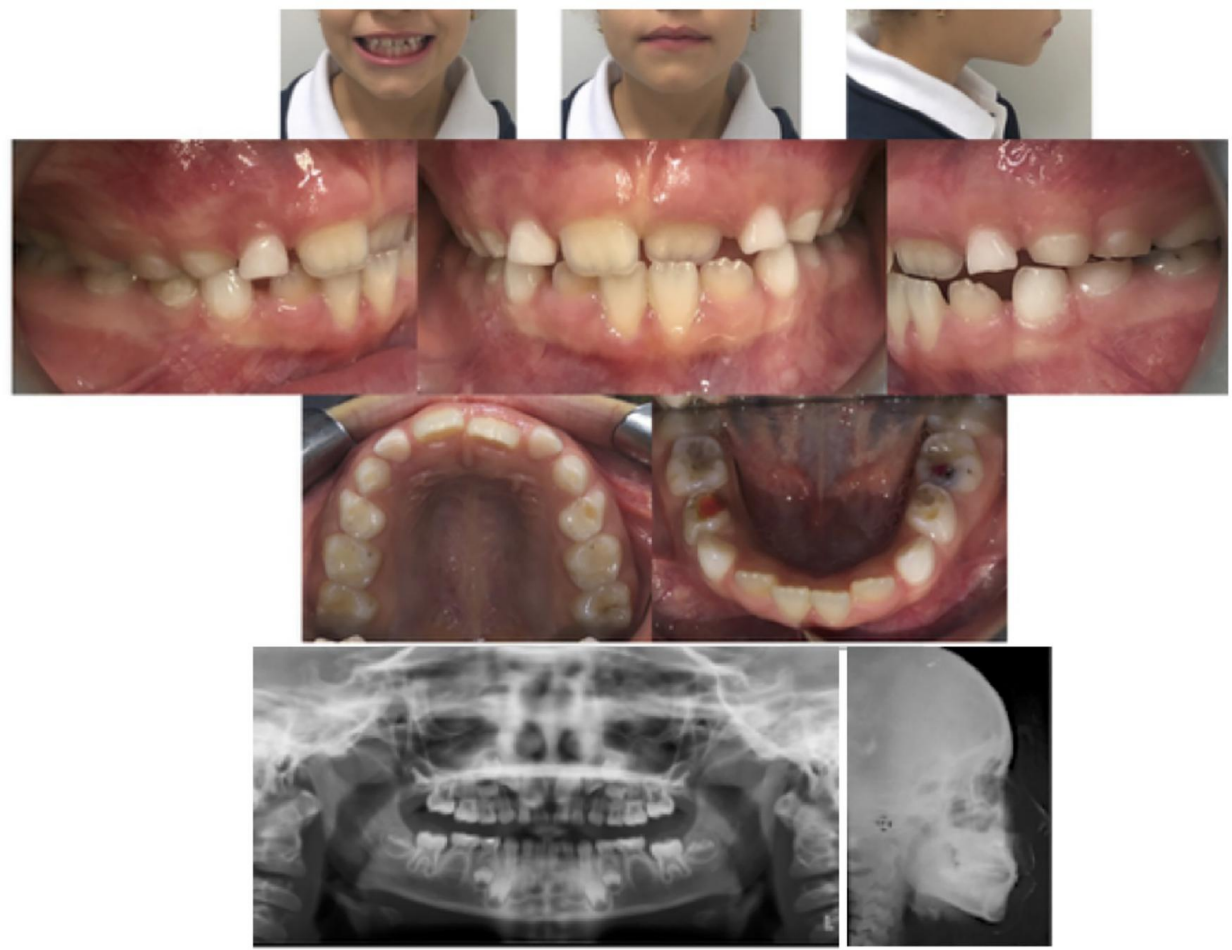
Pre-treatment records.

A 9-year-old female presented with Class I malocclusion in mixed dentition, crossbite of the upper left central incisor and slight crowding in the mandibular arch. She had a negative overjet (−1 mm) and a normal overbite. Although there was no hereditary tendency to Class III skeletal malocclusion, we did perform a cephalometric x-ray. The soft tissue analysis highlighted a regular profile, and frontal view did not present any facial asymmetry: indeed, the maxillary midline was coincident both with the mandibular one and with the face. The patient's malocclusion was attributed only to an altered eruption pattern of permanent incisors (Figure 1).

The objectives of the orthodontic treatment were:

1. to correct the inclination of maxillary and mandibular incisors;
2. to treat traumatic contact at frontal tooth level;
3. to achieve leveling and alignment of both arches, thus eliminating functional shift of the mandible.

**Figure 2:**
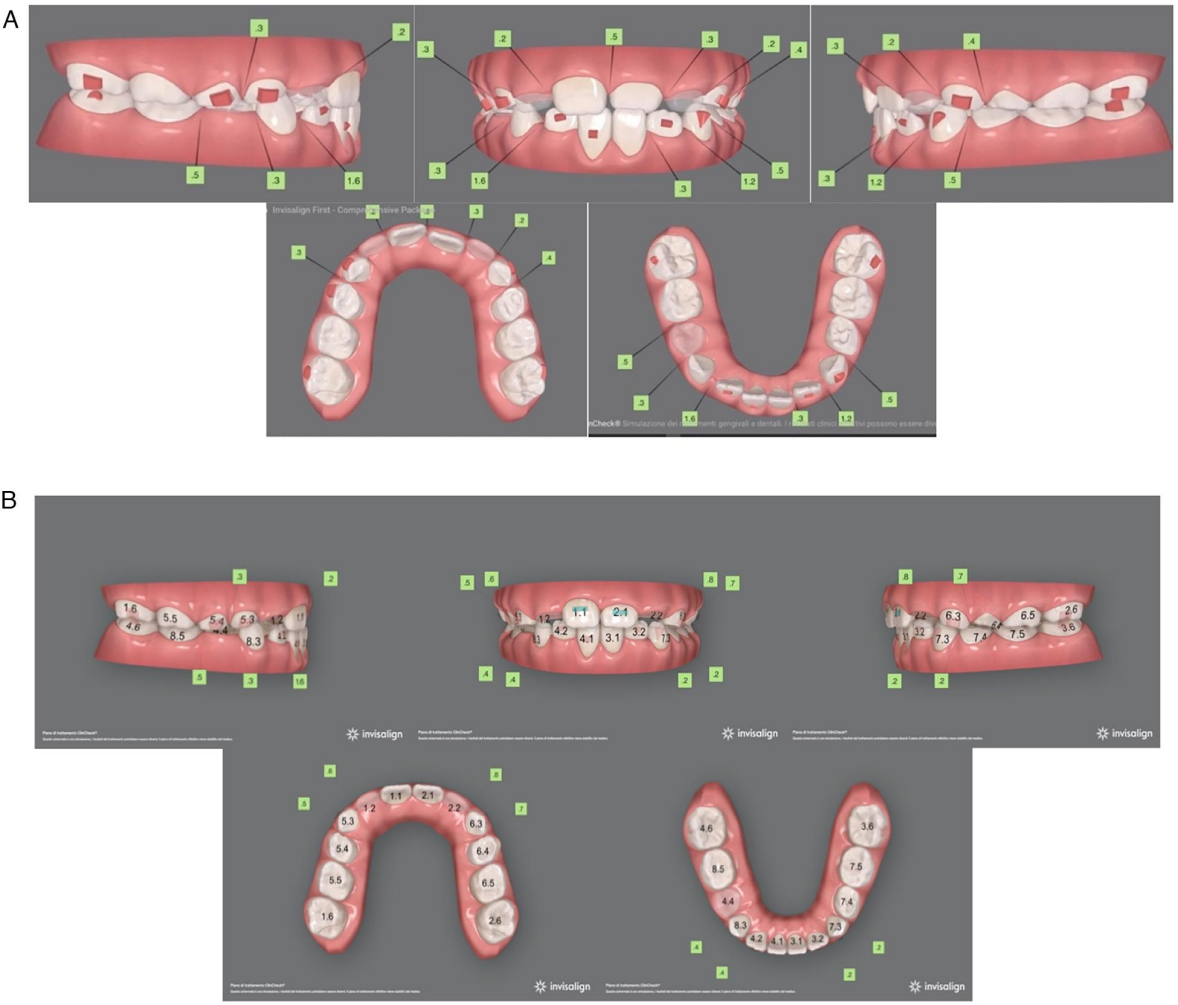
**(A)** Pre-treatment virtual projection. **(B)** Final virtual projection.

The virtual projection highlighted the possibility for proper resolution of all occlusal anomalies, correction of the overjet and overbite, and alignment of the upper anterior teeth. (Figures 2A,B)

Specifically, the treatment was based on 32 aligners per arch. Optimized attachments were planned at the level of posterior teeth to raise aligner stability and optimize forces on anterior teeth. Moreover, Power Ridges® were planned on the upper incisors to improve both force and torque (25, 26).

**Figure 3:**
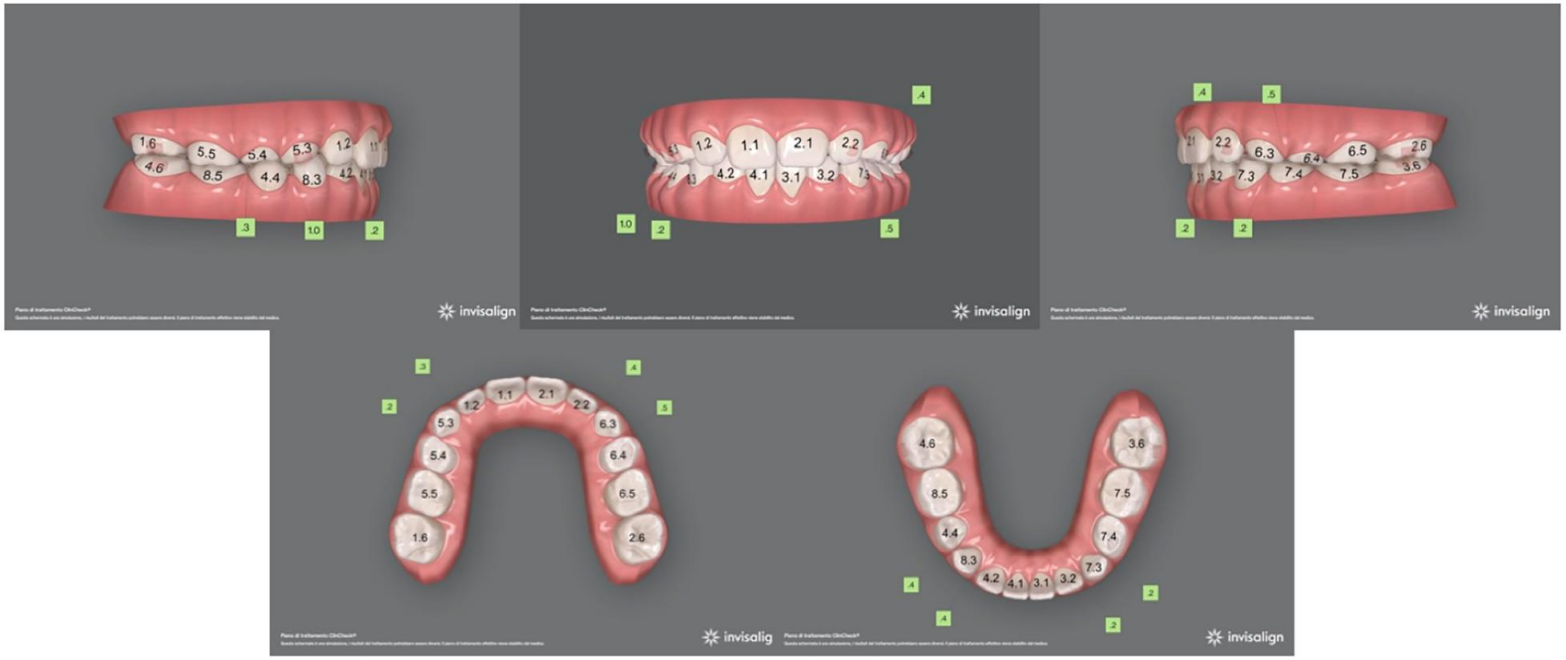
Virtual pre-refinement planning.

**Figure 4:**
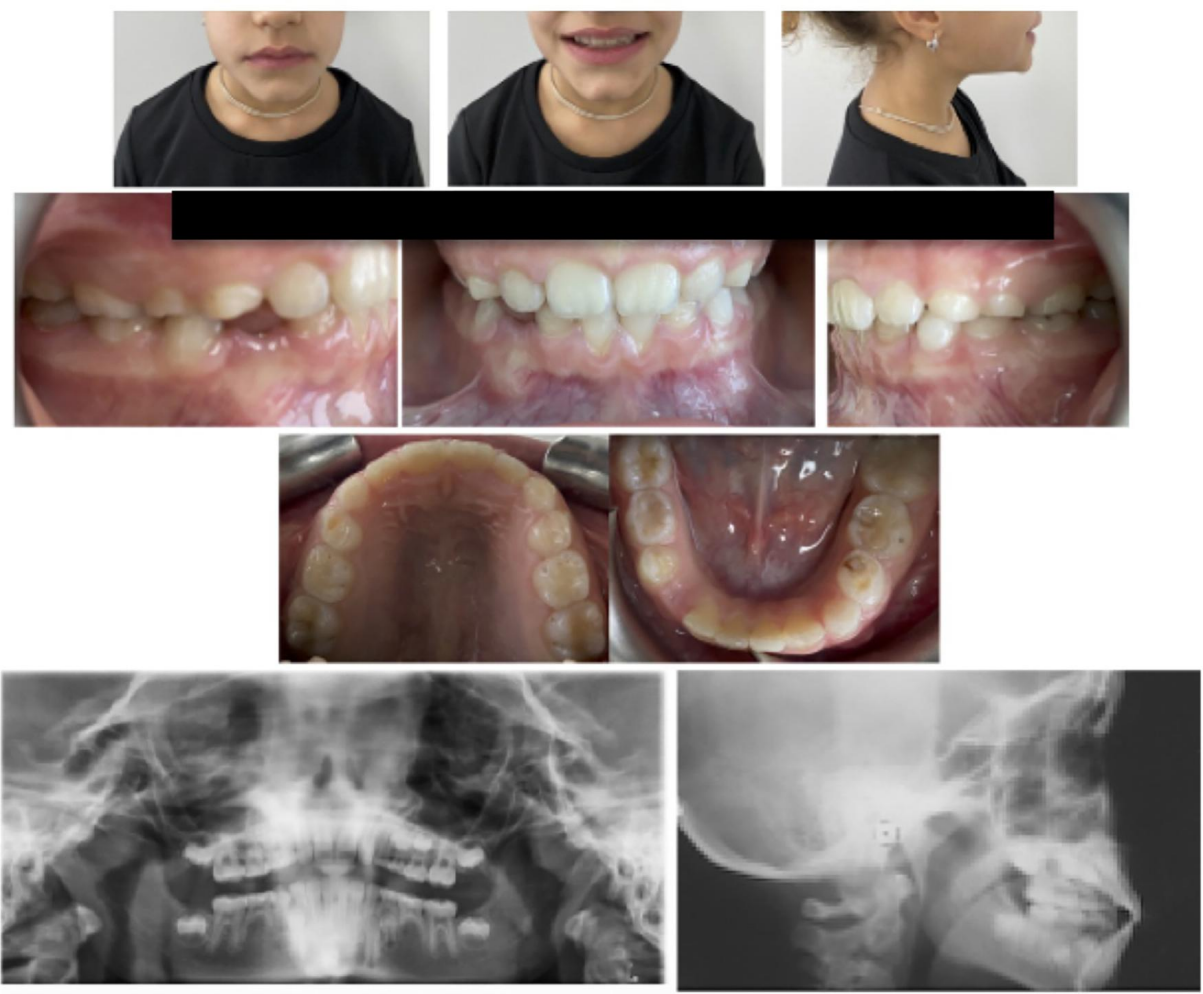
Post treatment records.

The upper arch was sequentially expanded and the upper first molars were adequately derotated. No interarch elastics were used. A 5-day-change protocol was adopted to achieve treatment goals. In terms of monitoring, the patient was seen every four weeks (4 to 5 pairs of aligners monthly) to check aligner fit, attachment stability, cooperation level, and dental care/hygiene. The initial phase lasted 6 months and 2 weeks. Complete malocclusion correction required 10 additional aligners and Class III elastics to balance occlusal contacts and improve the gingival profile on the upper incisors (Figure 3). Overall, full correction was achieved in 11 months. By the end of the treatment, proper overjet and overbite were established, while a good relationship between the maxilla and the mandible was maintained (Figure 4). In order to pursue outcome stability, the patient wore the last pair of aligners for another 4 months only at night time.

### Case report 2

**Figure 5:**
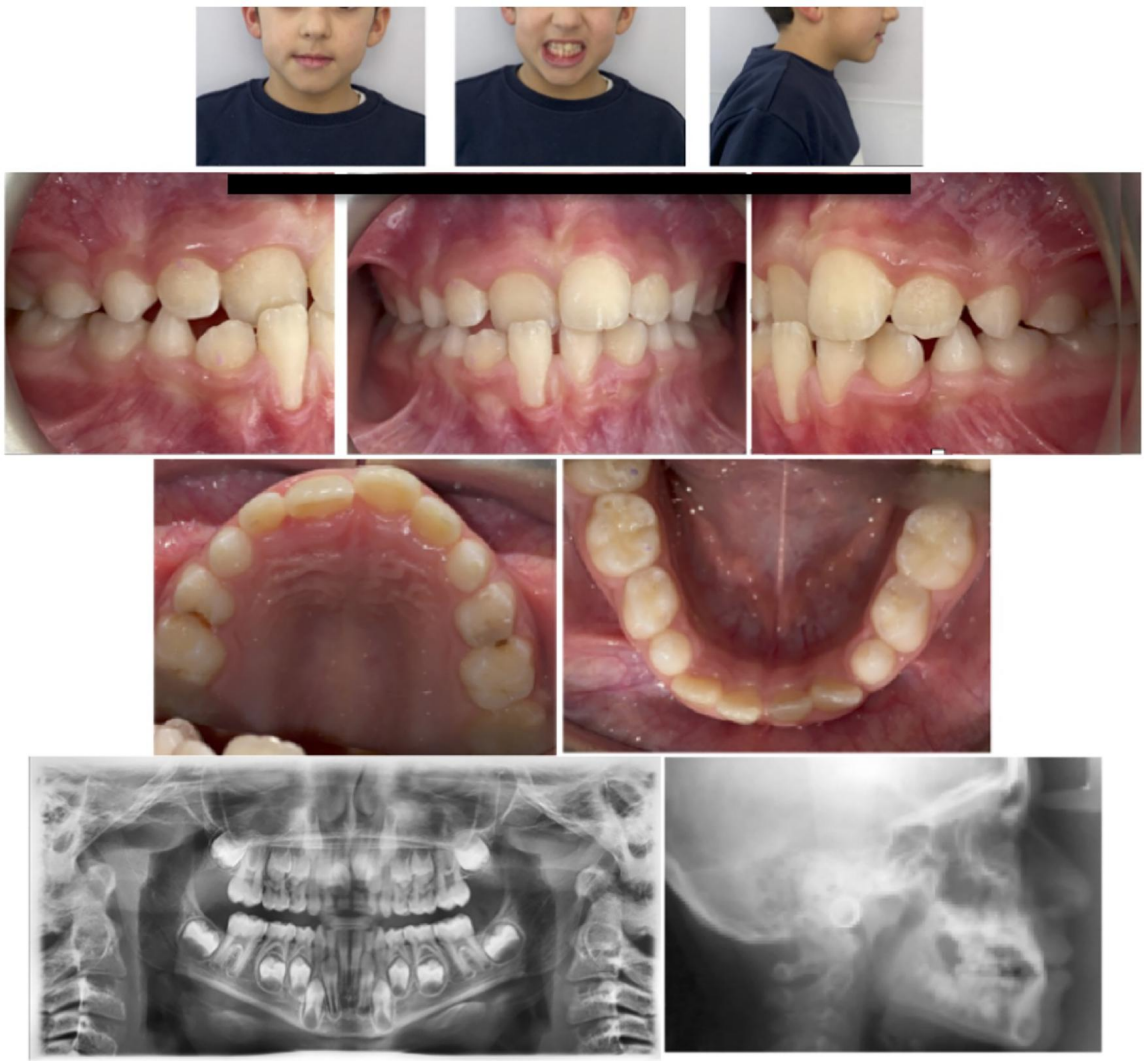
Pre-treatment records.

A 9-year-old male presented with an anterior crossbite of the upper right central incisor, in mixed dentition and dental Class I malocclusion. In the lower arch, a mild diastema between central incisors with an evident gum recession of 4.1 was present. The patient's soft tissue profile was slightly concave, despite being well balanced overall. No asymmetry was identified upon frontal observation, nor was Class III malocclusion familiarity reported. On the whole, the patient's dental conditions were acceptable, although gum recession may have been caused by occlusal dental trauma (Figure 5). Following assessment, malocclusion was attributed to an altered eruption pattern of permanent incisors. Hence, possible treatment options included clear aligner treatment, which was prioritized in accordance with parents.

**Figure 6:**
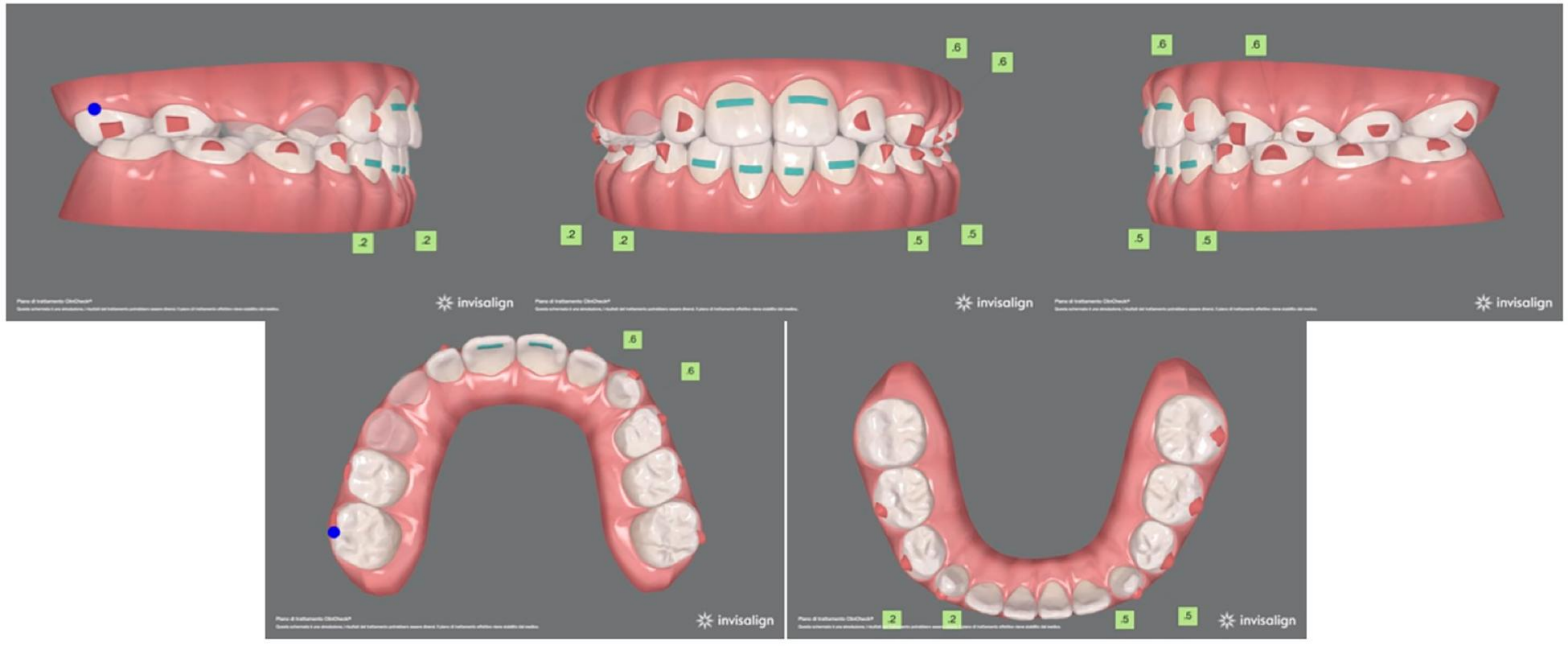
Initial virtual projection.

**Figure 7:**
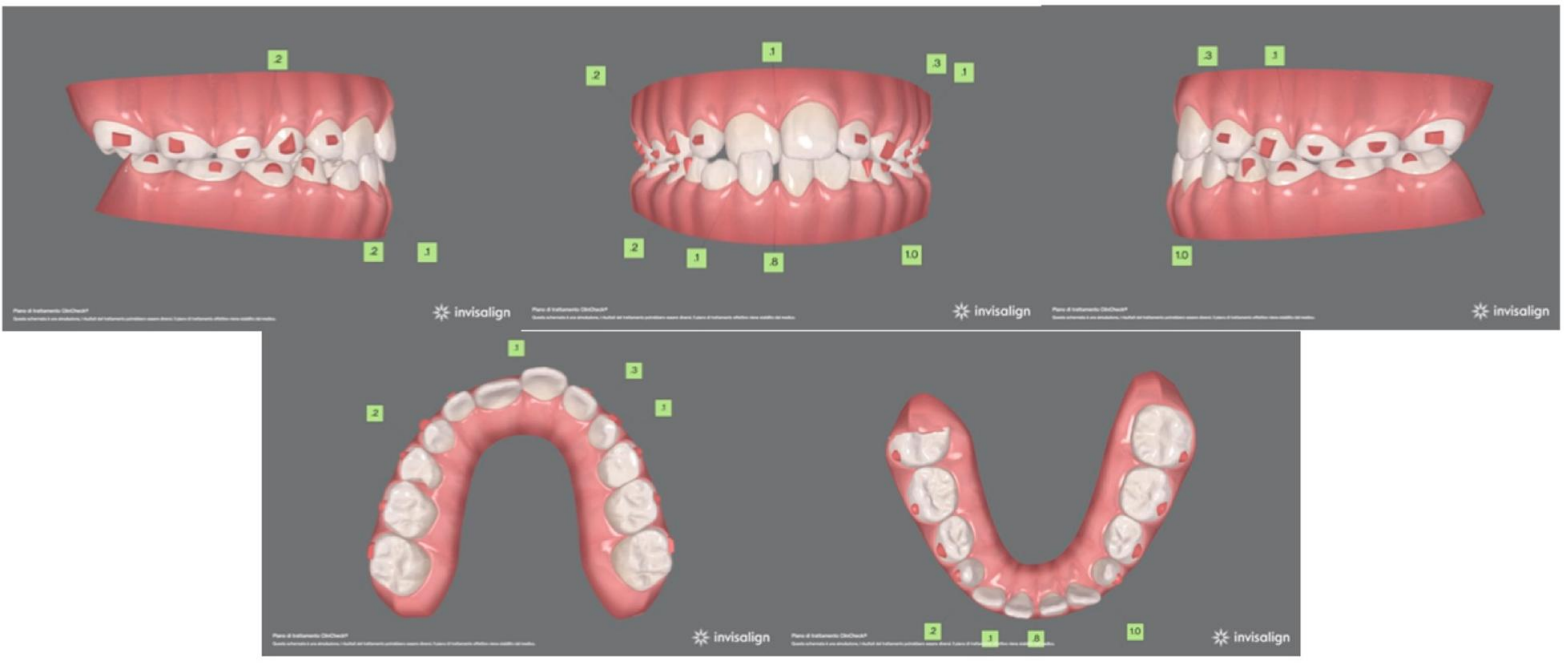
Final virtual projection.

**Figure 8:**
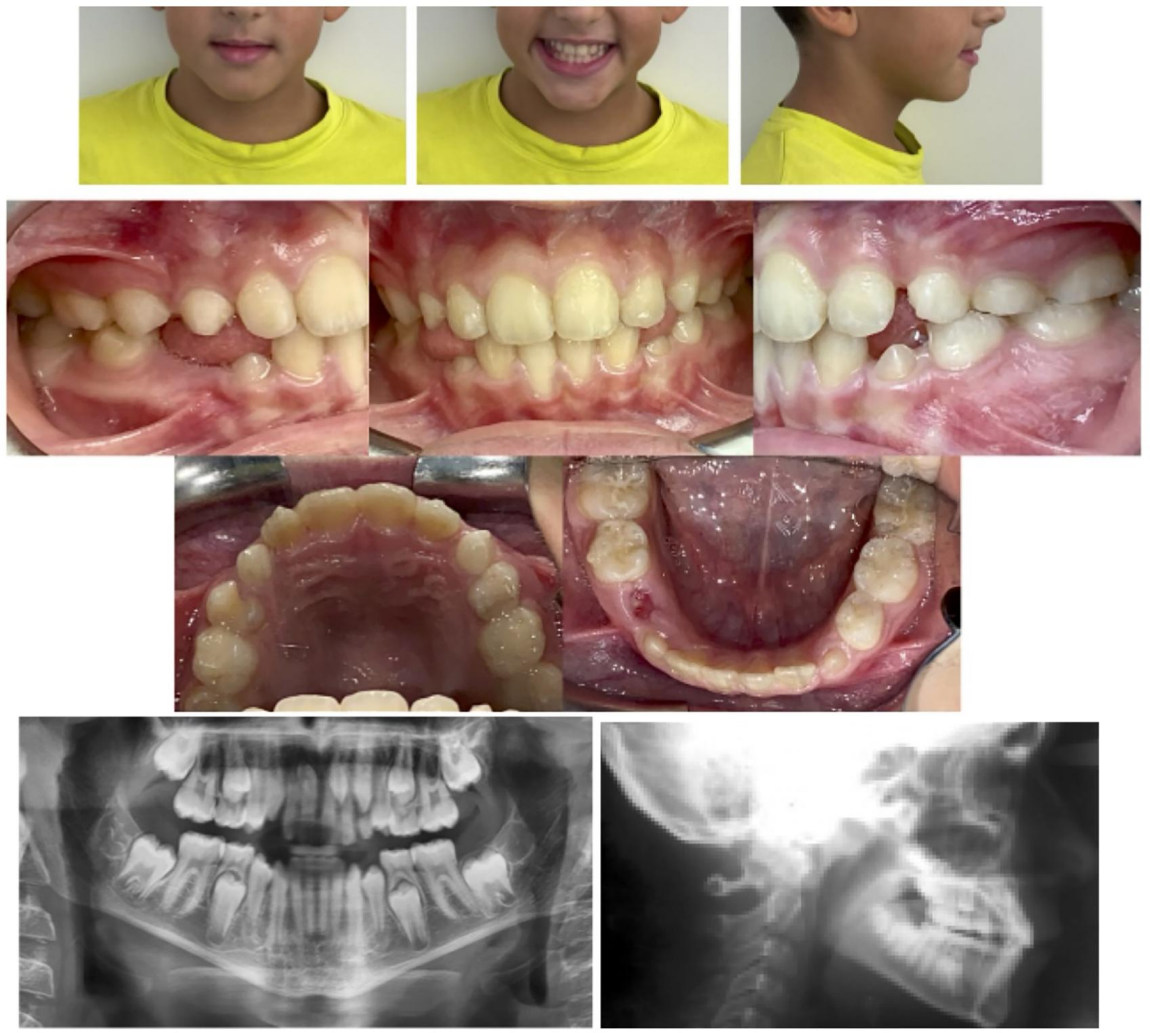
Post-treatment records.

The first treatment phase consisted of 35 aligners in the upper arch and 30 aligners in the lower one. Moreover, optimized attachments were placed on the posterior teeth to raise aligner stability and to compensate for the reduced clinical crown height. Additionally, Power Ridges® on central upper incisors were designed to improve front tooth torque (Figures 6, 7). As indicated to the patient, aligners were to be changed every 5 days. The patient was monitored on a monthly basis to check aligner fit and proper hygiene. On the other hand, the second treatment phase required further 15 aligners per arch. By the end of the overall 10-month treatment, the inclination of maxillary and mandibular incisors was properly settled. Regarding lower gum recession, it improved by 3 mm in terms of height and, alongside, the patient proved to be well motivated to maintain proper oral hygiene conditions (Figure 8). At the end of the active treatment, the last pair of aligners was prescribed as a night retainer for 6 additional months.

## Discussion

The aim of this study was to evaluate the effectiveness of clear aligners in treating growing patients with anterior crossbite. Literature suggests several treatment options for the correction of anterior crossbite in dental Class I and/or pseudo-Class III malocclusion cases, (2, 6, 14, 15) although, more recently, a number of clinicians have shown evidence of clear aligner therapy as a valuable option (22, 25, 26).

Moreover, it is noteworthy to consider how literature has also highlighted that other important aspects of early intervention should be evaluated in mixed dentition treatment. These include cost-benefit (23) and possible complications during treatment (displacement, breakage, and loss of appliances) (36), in addition to other variables, such as the perception of pain and discomfort associated with treatment (37). However, given the limited literature references to individual perception, it is herein suggested to carry out future research aimed at evaluating the patient's perception of the physical and psychological effects of aligner-based orthodontic protocols (38).

Indeed, the aforementioned technique is typically well tolerated by young patients and allows them to comfortably participate in daily and social activities without major concerns, thus improving their treatment experience (39, 40).

More importantly, the use of clear appliances also prevents dental decalcifications during orthodontic treatment, as clear aligners favor suitable oral hygiene (23–25). However, clear aligner therapy is significantly based on patient compliance, whose lack would lead to treatment failure. Clear aligner treatment is typically easily accepted by patients who are either reluctant to or may feel distressed by fixed orthodontic appliances. Finally, the shorter treatment duration compared to conventional device-based treatments favors positive feedback from both patients and caregivers (23, 25, 26).

Nowadays, the digital aligner protocol workflow drives a virtual orthodontic plan with the possibility of predicting dental movements in both arches for a better, easier, and more rapid correction of the relationship between upper and lower incisors. The effectiveness and efficacy of this treatment allow to achieve dental movements more precisely (25, 26).

In anterior crossbite cases, correction is achieved through palatal-vestibular tipping movements of the incisors, ensuring proper torque control and arch disclosure.

Tipping movements are highly predictable. According to Kravitz et al., crown tipping movements are among the most predictable ones in aligner-based therapy, accounting for a mean accuracy of 47% (41). Additionally, Lombardo et al. reported that the mean predictability of vestibulolingual tipping is 72.9% (35). More recently, an international modified Delphi consensus highlighted that aligners are effective at performing tipping movements with a 72% consensus among the experts involved in the study (42).

With the aim of improving lingual root torque expression, Power Ridges® were introduced by Align Technology in 2009 (43). By using these specific features, the performance of aligners is commonly thought to improve further (44), optimizing torque control in cases of anterior crossbite. However, recent literature reports that there are no significant differences in outcomes between patients treated with and without Power Ridges® (45–47).

Optimized anchorage attachments can be placed on posterior teeth, raising aligner stability and, in turn, improving aligner biomechanics. On the other hand, in case of anterior crossbite, tipping movements do not necessarily require the use of dedicated attachments.

Compared to conventional fixed appliances, using aligners for anterior crossbite correction may be more suitable considering that the thickness of the devices themselves allows for a natural disarticulation on the arches. Consequently, vertical dimension is raised without the aid of other devices such as bite turbos, posterior bite raisers or bite plates that are used in conventional orthodontics. If necessary, occlusal attachments on molars could be strategically planned in the virtual set-up to promote greater posterior bite opening (48).

To our knowledge, the existing literature does not include thorough studies focusing on the effectiveness of clear aligners for pseudo-Class III treatment of growing patients. Despite this type of treatment is described in the literature, it is mostly in the form of case reports or case series (26, 49).

Although the topic is innovative and the results achieved are encouraging, two major limitations of the herein study shall be considered: limited sample size and short-term observation. Indeed, further investigation and research focusing on a larger sample size and longer-term observation would be ideal.

## Conclusion

Clear aligners are an aesthetic and more comfortable alternative to traditional fixed or removable orthodontic devices that can resolve anterior crossbite in mixed dentition. The results from the study conducted demonstrated the complete correction of the malocclusion across the 18-patient sample, evidencing a statistically significant increase of all the examined variables (OJ, AP, IC, SNA, SNB, ANB, and U1-NA).

According to our research, clear aligner treatment may represent a valid clinical approach in anterior crossbite correction in dental Class I and/or pseudo-Class III malocclusion cases when combined with good patient compliance (50). Moreover, one shall highlight the importance of proper diagnosis in order to obtain the best achievable outcome.

However, our study did reflect some constraints, as small sample size and limited observation period. Therefore, evaluating post-correction stability and long-term result maintenance would be important by means of further clinical research.

## Data Availability

The raw data supporting the conclusions of this article will be made available by the authors, without undue reservation.
